# Mr. Ballard, on a Particular Species of Ulcer

**Published:** 1800-11

**Authors:** John Ballard

**Affiliations:** Surgeon, Royal Navy. Portsmouth


### *To the Editors of the Medical and Phyfical Journal.*

GENTLEMEN,

IF you think the following remarks on the inveterate fpecies of Ulcer, which has lately been fo deftructive in his Majefty's fhips, worthy a place in your valuable Journal, the inferting it will confer a favour on,

GENTLEMEN,

Your very humble fervant,

JOHN BALLARD, Surgeon,

Royal Navy.

*Portsmouth,*

*Auguft* 12, 1800.

---

I entered into the navy in March, 1798, with an appointment to his Majefty's fhip Triumph, then commanded by Captain William Effington; on entering on my profeffional duty, I was ftruck with the appearance of the ulcers, which had been for fome time under the care of my predeceffors; they were very different from the appearance of any ulcers I had ever feen before, either in private practice, or during my attendance at the General Hofpital near Birmingham; I was therefore refolved

ſolved to pay very peculiar attention to them, and obſerve their progreſs, for I really predicted in my own mind, that we ſhould have ſome trouble in the management of them. I collected what information I could reſpecting them from my profeſſional friends on board, and was informed, that a man with a very ill-conditioned ulcer had been received on board the Triumph from the guard-ſhip at Spithead, and from the date of his introduction into the ſhip, they had obſerved ſome alteration in the complexion of the ſores; but it did not ſeem to be ſo apparent to them as to me, who had ſo lately come from the ſhore; I did not, however, loſe ſight of my own opinion; it is true, two or three caſes healed firmly, but notwithſtanding, we could eaſily perceive, that every ſucceſſive caſe became more and more inveterate.—The Triumph ſailed with Lord Bridport on the 12th of April, 1798, with ſeveral bad caſes of ulcers in the liſt of ſick; we returned to Torbay about a month afterwards, with a conſiderable increaſe of theſe caſes; in ſhort, the ſituation appropriated for the reception of the ſick on board of a ſhip, was completely filled. Dr. Trotter, the phyſician to the fleet, was applied to, and ſome of the worſt caſes were ſent on ſhore; ſtill, too many were retained. We did not remain long at Torbay; and the Triumph's next cruiſe was not leſs than three months, during which time we were ſadly haraſſed with increaſing numbers of theſe deſperate caſes. Mr. Thomas Moffatt, the judicious ſurgeon of the Triumph, uſed every exertion to overcome this deſtructive complaint, and every external means that afforded even a gleam of hope was tried; the fumes of nitric acid were uſed three weeks with unremitting attention, but without any permanent advantage reſulting, and it was relinquiſhed on that account. The Triumph returned to Plymouth with a great number of theſe ulcers, and the greater part of them was ſent to the Naval Hoſpital there.

To a cruiſe on the coaſt of Ireland theſe baneful attendants followed us, now accompanied with alarming fever, which did not appear before, requiring the moſt active remedies of inflammation, which generally terminated it in three or four days, leaving the patient exceſſively reduced. Opium was then uſed with great advantage, by ſuſpending the irritation occaſioned by the continual abſorption of putreſcent particles into the ſyſtem. I am indebted to a very reſpectable phyſician in Birmingham, for an idea reſpecting the nature of the fever and inflammation attending theſe caſes; and he calls the fever a ſpecies of typhus, and the inflammation a ſpecies of eryſipelas, which accord perfectly with my ideas. I ſhould not have ventured to give this account to the public, for I am a very young ſurgeon; furgeon; but I am actuated by very feducing motives; 1ft. In juftice to my worthy and much efteemed preceptor from whom I received the firft and the only rudiments of my profeffion I at prefent poffefs, and partly on account of fome information I have received fince my return from the Weft Indies, from a very refpectable practitioner in the navy, that thefe ulcers are ftill in exiftence; and having myfelf been an actual obferver of their deftructive influence, I cannot, in confcience, withold what I think the only means of eradicating them from the navy. The practitioner above alluded to, has told me, that his fhip was free from any fuch cafes, "till the introduction of fome bad ones from a fhip in the Channel fervice, and in vain did he endeavour to eradicate them by external means. Thus circumftanced, he refolved to fend every cafe, however flight, out of the fhip, and totally deftroy every thing that had been ufed in dreffing them," viz. fponge, lint, &c. and it is very pleafing for me to remark, that this practice was attended with the happieft effect: I have the teftimony of himfelf, and my own actual obfervation, that he has not, at prefent, one folitary cafe of bad ulcer under his care. From thefe evidences, I moft fincerely recommend a fimilar mode of proceeding in every fhip in his Majefty's fervice, in which thefe cafes exift, for I am convinced that no other *means will be effectual in banifhing them from the Navy.*

